# Supplementary material for: A web application for the design of multi-arm clinical trials
Source: BMC Cancer. 2020 Jan 31;20:80. doi: 10.1186/s12885-020-6525-0 (PMC6995188; doi:10.1186/s12885-020-6525-0)
Supplement: Supplementary file 1 — Additional file 1 PDF report. A copy of the PDF report generated by clicking the Generate report button in the web application, for the input parameters shown in Fig. 1. [file 12885_2020_6525_MOESM1_ESM.pdf]

Fixed-sample multi-arm clinical trial design for a Bernoulli distributed primary outcome

20/06/2019

Design setting

The trial will be designed to compare K experimental treatments to a shared control arm. Response X_{ik}, from patient $i = 1, \dots, n_k$ in arm $k = 0, \dots, K$, will be assumed to be distributed as $X_{ik} \sim Bern(\pi_k)$. Then, the hypotheses to be tested will be:

$$H_k : \tau_k = \pi_k - \pi_0 \leq 0,\ k = 1, \dots, K.$$

The *global null hypothesis*, H_G, will be:

$$\pi_0 = \cdots = \pi_K.$$

The *global alternative hypothesis*, H_A, will be:

$$\pi_1 = \cdots = \pi_K = \pi_0 + \delta_1.$$

The *least favourable configuration* for experimental arm k, LFC_k, will be:

$$\pi_k = \pi_0 + \delta_1,\ \pi_1 = \cdots = \pi_{k-1} = \pi_{k+1} = \cdots = \pi_K = \pi_0 + \delta_0.$$

The *least favourable configuration* for experimental arm k, LFC_k, will be:

$$\tau_k = \delta_1,\ \tau_1 = \cdots = \tau_{k-1} = \tau_{k+1} = \cdots = \tau_K = \delta_0.$$

Here, δ_1 and δ_0 are *interesting* and *uninteresting* treatment effects respectively.

Inputs

The following choices were made:

- $K = \mathbf{2}$ experimental treatments will be included in the trial.
- A significance level of $\alpha = \mathbf{0.15}$ will be used, in combination with **Dunnett's correction**.
- The response rate in the control arm will be assumed to be: $\pi_0 = \mathbf{0.3}$.
- The **marginal power for each null hypothesis** will be controlled to level $1 - \beta = \mathbf{0.8}$ under **each of their respective least favourable configurations**.
- The interesting and uninteresting treatment effects will be: $\delta_1 = \mathbf{0.15}$ and $\delta_0 = \mathbf{0}$ respectively.
- The target allocation to each of the experimental arms will be: **the same as the control arm**.
- The sample size in each arm **will not** be required to be an integer.
- Plots **will** be produced.

Outputs

- The total required sample size is: $N = \mathbf{293.963}$.
- The required sample size in each arm is: $(n_0, \dots, n_K) = \mathbf{(97.988, 97.988, 97.988)}$.
- Therefore, the realised allocation ratios to the experimental arms are: $(r_1, \dots, r_K) = \mathbf{(1, 1)}$.
- The maximum familywise error-rate is: **0.15**.
- The **minimum marginal power** is: **0.8**.
- The following critical threshold should be used with the chosen multiple comparison correction: **0.087**.

pi0	pi1	pi2	FWERI1	FWERI2	FWERII1	FWERII2
0.3	0.30	0.30	0.1499712	0.0273161	0.0000000	0.0000000
0.3	0.45	0.45	0.0000000	0.0000000	0.3190556	0.0832455
0.3	0.45	0.30	0.0879297	0.0000000	0.2000000	0.0000000
0.3	0.30	0.45	0.0879297	0.0000000	0.2000000	0.0000000

pi0	pi1	pi2	Pdis	Pcon	P1	P2
0.3	0.30	0.30	0.1499712	0.0273161	0.0886437	0.0886437
0.3	0.45	0.45	0.9167545	0.6809444	0.7988494	0.7988494
0.3	0.45	0.30	0.8024691	0.0854606	0.8000000	0.0879297
0.3	0.30	0.45	0.8024691	0.0854606	0.0879297	0.8000000

pi0	pi1	pi2	PHER	FDR	pFDR	FNDR	Sens	Spec
0.3	0.30	0.30	0.0886437	0.1499712	1.0000000	0.0000000	0.0000000	0.9113563
0.3	0.45	0.45	0.0000000	0.0000000	0.0000000	0.3190556	0.7988494	0.0000000
0.3	0.45	0.30	0.0439648	0.0451994	0.0563254	0.1012345	0.8000000	0.9120703
0.3	0.30	0.45	0.0439648	0.0451994	0.0563254	0.1012345	0.8000000	0.9120703

Plots

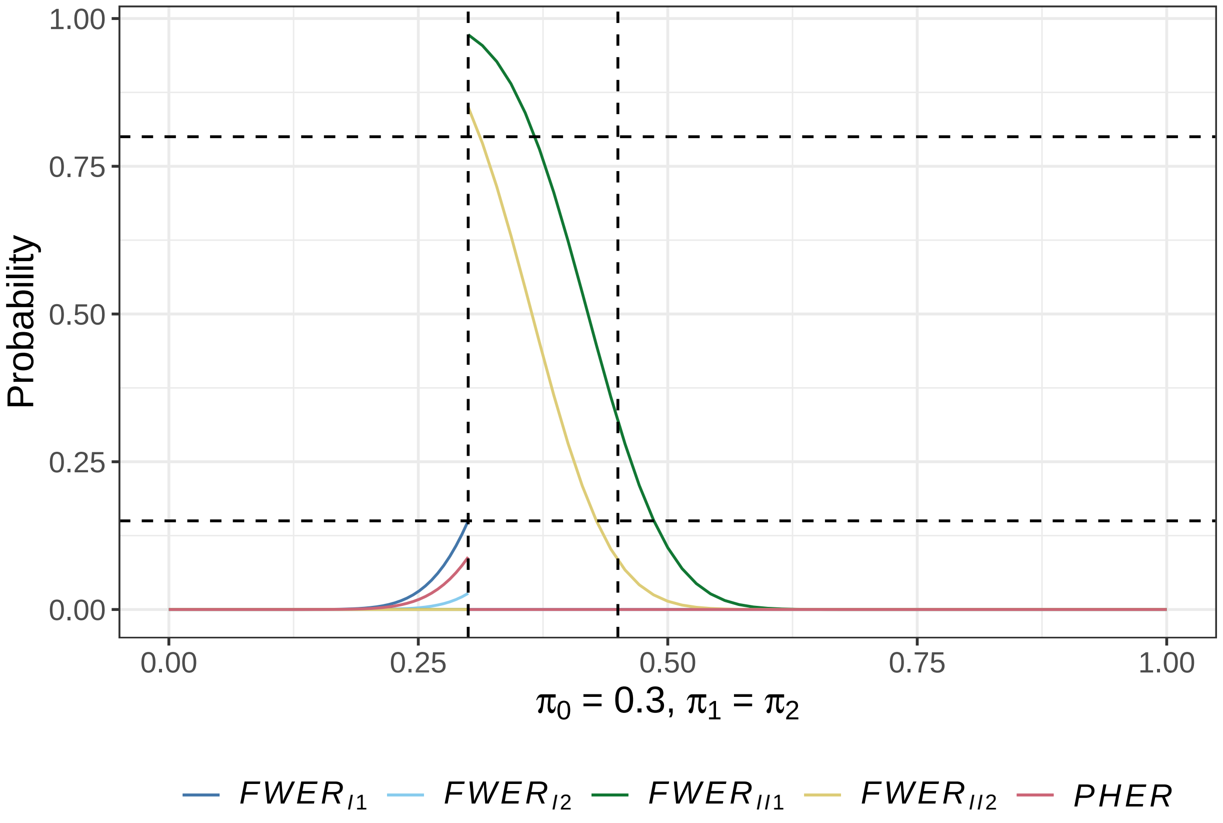

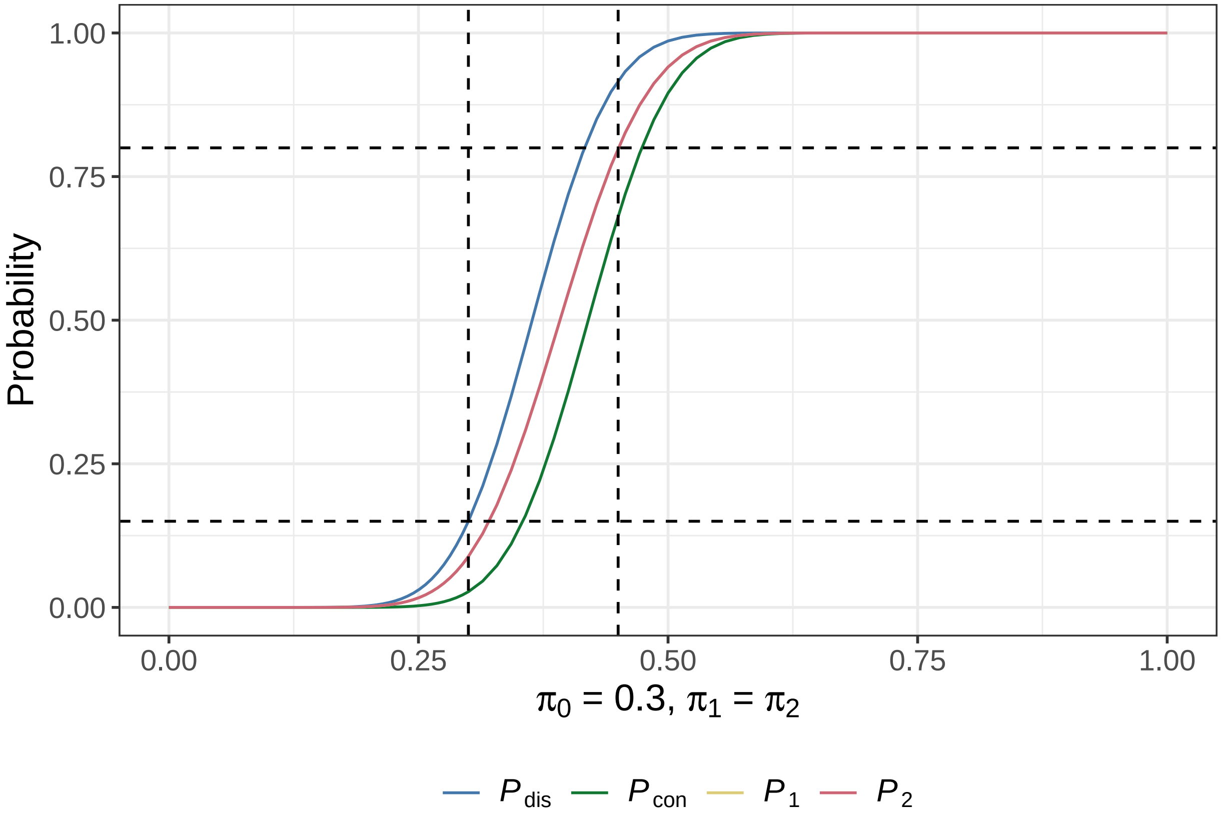

1.00
0.75
0.50
0.25
0.00
Probability
0.00
0.25
0.50
0.75
1.00
$\pi_0 = 0.3, \pi_1 = \pi_2$
P_{dis}
P_{con}
P_1
P_2

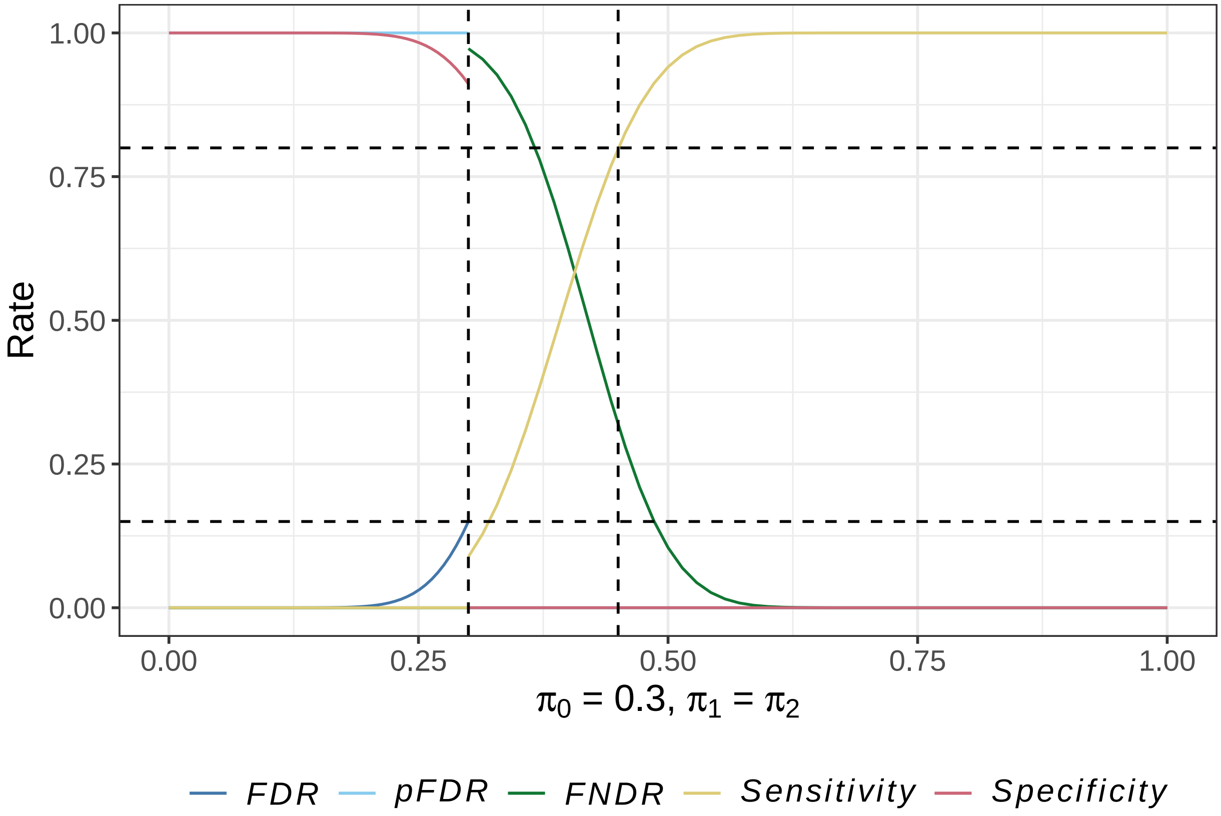

1.00
0.75
0.50
0.25
0.00
Rate
0.00
0.25
0.50
0.75
1.00
$\pi_0 = 0.3, \pi_1 = \pi_2$
FDR
pFDR
FNDR
Sensitivity
Specificity

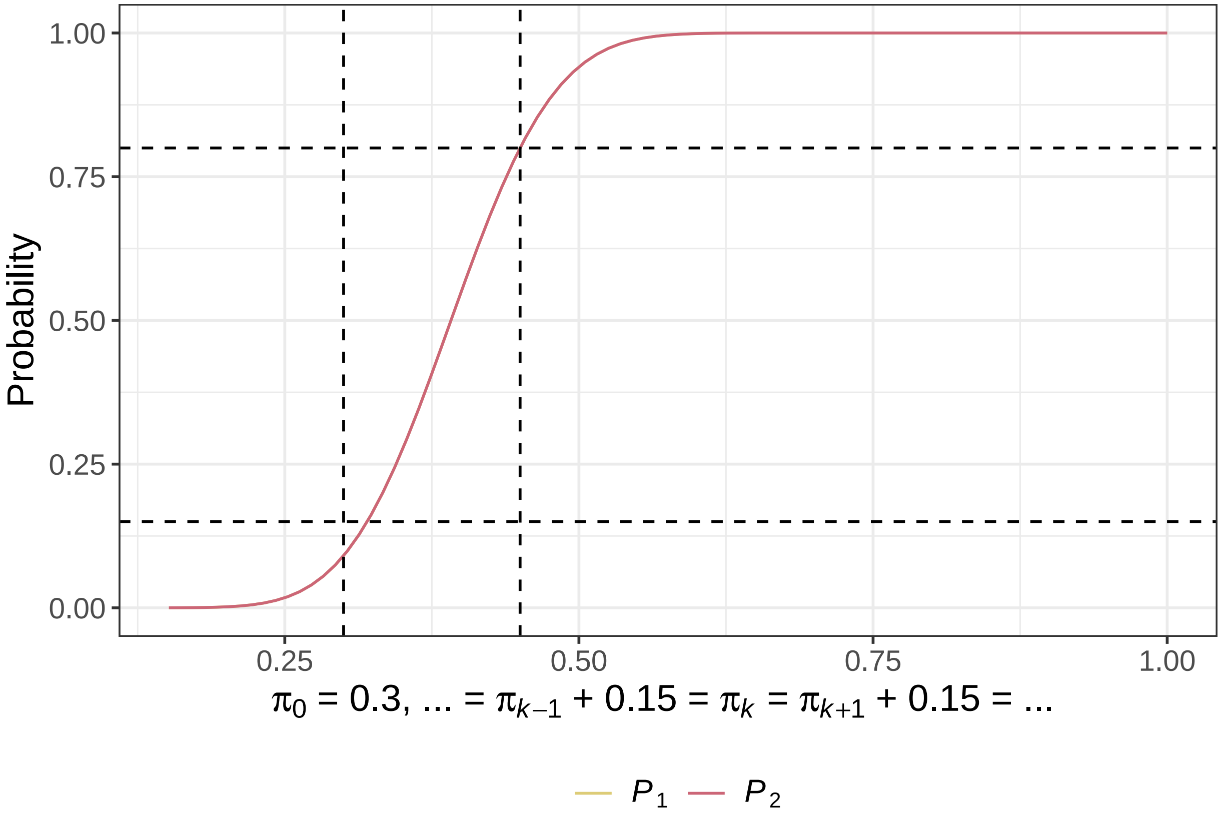

Probability
1.00
0.75
0.50
0.25
0.00
0.25
0.50
0.75
1.00
π_0 = 0.3, ... = π_{k-1} + 0.15 = π_k = π_{k+1} + 0.15 = ...
P_1
P_2
